# Robotic magnetic navigation-guided pulmonary vein isolation in an atrial fibrillation patient with dextrocardia situs inversus: techniques and potential advantages

**DOI:** 10.1186/s12872-023-03308-6

**Published:** 2023-05-20

**Authors:** Qingzhi Luo, Yun Xie, Liqun Wu, Qi Jin

**Affiliations:** Department of Cardiovascular Medicine, Ruijin Hospital, Shanghai Jiao Tong University School of Medicine, No. 197, Ruijin Er Road, Shanghai, 200025 China

**Keywords:** Atrial fibrillation, Dextrocardia situs inversus, Robotic magnetic navigation, Catheter ablation, Case report

## Abstract

**Background:**

Dextrocardia with situs inversus (DSI) is a very rare congenital anomaly. Catheter manipulation and ablation of atrial fibrillation (AF) in patients with this anatomical variant is challenging for the operators. This case report presents a safe and effective AF ablation guided by the robotic magnetic navigation (RMN) system in combination with intracardiac echocardiograhy (ICE) in a patient with DSI.

**Case presentation:**

A 64-year-old male with DSI was referred for catheter ablation of symptomatic, drug-refractory paroxysmal AF. One transseptal access was achieved via the left femoral vein under the guidance of ICE. The three-dimensional reconstruction of the left atrium and the pulmonary veins (PVs) were performed by the magnetic catheter using the CARTO and the RMN system. Then, the electroanatomic map and pre-acquired CT images were merged. Finally, bilateral circumferential ablation lines were delivered around the ipsilateral PV ostia to achieve complete PV isolation (PVI).

**Conclusions:**

This case demonstrates that AF catheter ablation under the guidance of the RMN system using ICE is feasible and safe in a patient with DSI. Moreover, the combination of these technologies broadly facilitates treatment of patients with complex anatomy, while reducing the risk of complications.

## Background

Dextrocardia is a very rare congenital anomaly, with an incidence of 1–2 cases per 20,000 people in the general population, and one-third of these patients have dextrocardia with situs inversus (DSI), characterized by mirror image of normal viscera-heart arrangement [1]. In general, patients with DSI have a low incidence of congenital cardiac abnormalities and are associated with an expected long life expectancy. Atrial fibrillation (AF) is one of the most common types of arrhythmias and pulmonary vein isolation (PVI) is the cornerstone of ablation for the treatment of patients with symptomatic and drug-refractory AF. Computed tomography (CT) geometry reconstruction shows a mirror image between DSI and normal heart, with the interatrial septum (IAS) in DSI patients rotating 90° counterclockwise on the sagittal plane [2]. Thus, transseptal puncture (TSP), catheter manipulation and ablation procedures should be performed contrary to normal in patients with DSI, and two-dimensional information provided by X-ray images is insufficient, which may present challenges for the operators. Robotic magnetic navigation (RMN) is a technique using magnetic field to control the mapping and ablation catheter inside the heart. The main advantages of RMN-guided catheter ablation are offering precise and omni-directional catheter navigation with superior reach, reduced fluoroscopy exposure, and an excellent safety profile [3]. In addition, the utilization of three-dimensional (3D) electroanatomical mapping (EAM) system and intracardiac echocardiography (ICE) can provide more real-time information and reduce the risk of complications during TSP and subsequent ablation procedures in AF patients with this anatomical variant. This case report presents a safe and effective AF ablation guided by RMN system in combination with ICE in a patient with DSI.

## Case presentation

**Fig. 1:**
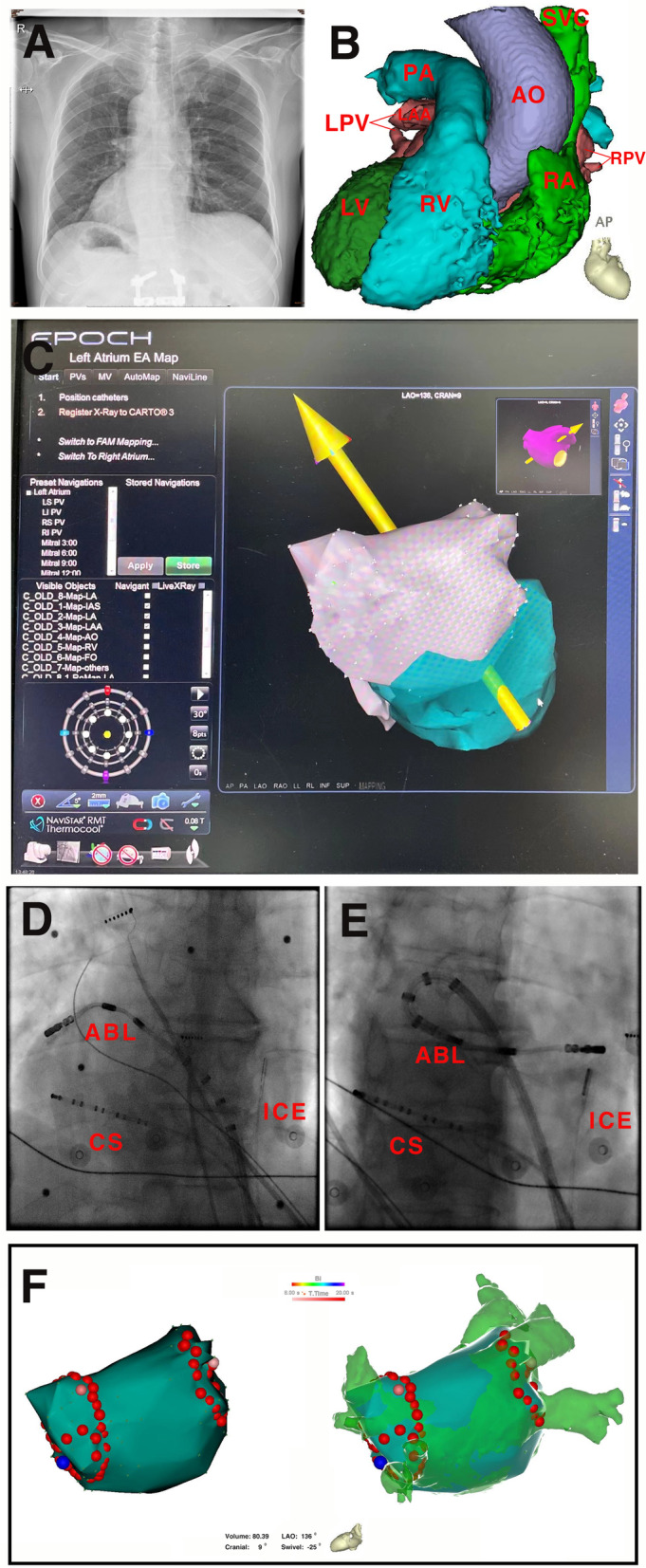
Panel **A** Chest radiograph reveals DSI with left-sided liver and right-sided stomach. Panel **B** Cardiac CT scan shows the inferior and superior vena cava connecting to the RA on the left side, and the structure and position of all four cardiac chambers presents a mirror image of a normal heart.Panel **C** The 3D anatomical reconstruction of the LA acquired with ICE is merging with reconstructions by the ablation catheter in the RMN system. Panel **D** The magnetic ablation catheter is positioned in the left inferior PV in RAO view, with a multipolar electrode catheter in CS and ICE catheter at mid-RA. Panel **E** The magnetic ablation catheter along with the steerable sheath forms a large loop in LA when ablating right PV in RAO view. Panel **F** The 3D electroanatomical reconstruction of the LA and PVs is created using the CARTO and RMN system, merging with pre-acquired CT image. Red dots indicate the ablation line around the bilateral PVs

A 64-year-old male with 3-year paroxysmal AF was admitted into our hospital. Episodes of AF were highly symptomatic, appeared two to three times per month, lasted for one to two hours and were terminated by beta blocker or propafenone. Chronic antiarrhythmic therapy with class IC drugs and beta blocker was ineffective for AF prevention. He had a history of hypertension and diabetes mellitus. The baseline rhythm was sinus on electrocardiogram at admission. Thus, the patient was referred for catheter ablation of AF according to current guidelines. Preoperative transthoracic echocardiography revealed DSI without associated cardiac anomalies. The structure, and position of all four cardiac chambers present a mirror image of the normal heart, with L-looped ventricles and inverted great arteries, and the left atrium (LA) diameter at 41 mm. Chest radiograph and cardiac contrast-enhanced CT scan showed mirror images of the normal visceral position (left-sided liver and right-sided stomach) (Fig. 1A and B). Transesophageal echocardiography showed no intracardiac thrombi. An uninterrupted anticoagulation strategy with rivaroxaban was adopted peri-procedure. After obtaining written informed consent, the procedure was performed under local anesthesia with lidocaine and deep sedation with midazolam and fentanyl. Femoral vein (FV) access was established with two entry points on the right side and one on the left side. A multipolar catheter (IBI, St. Jude Medical Inc., St. Paul, MN) was inserted into the right FV, manipulated, and rotated into the coronary sinus (CS) under right anterior oblique (RAO) 30° view. An ICE catheter (SoundStar, Biosense Webster Inc. Irvine, CA) was advanced via the right FV and placed at the level of mid-right atrium (RA) to create an anatomic map of the LA (Fig. 1C). Next, an 8.5 F transseptal long sheath (Fast-Cath SL1, St. Jude Medical Inc., St. Paul, MN) with a Brockenbrough needle (BRK, St. Jude Medical Inc., St. Paul, MN) was introduced via the left FV and maneuvered into the superior vena cava, then pulled back slowly into the IAC and fossa ovalis clearly displayed by rotating the ICE catheter, and then tented into the LA with counterclockwise torque (8 o’clock position). On saline injection, a bubble visualized by ICE was used to verify successful TSP. Next, we replaced the 8.5 F SL1 sheath with a VIZIGO (Biosense Webster Inc. Irvine, CA) bi-directional guiding sheath in the LA. A 3.5 mm tip irrigated magnetic catheter (NaviStar™ RMT ThermoCool™, Biosense Webster Inc. Irvine, CA) was introduced into the LA through the steerable sheath and we performed 3D EAM of the LA and pulmonary veins (PVs) using the CARTO system (Biosense Webster Inc. Irvine, CA) and directing the catheter with the RMN Niobe ES system (Stereotaxis Inc., St. Louis, MO). Subsequently, the 3D reconstruction of the LA and previously acquired CT images were merged. Finally, bilateral circumferential ablation lines were delivered around the ipsilateral PVs ostia to achieve complete PVI (Fig. 1D, E, F). Radiofrequency (RF) energy was delivered with a power up to 40W, temperature limit set at 43 ℃, and an irrigation flow rate of 17 ml/min. The total procedural time, fluoroscopic time and RF time in this patient were 105 min, 6.3 min and 28.3 min, respectively. No complications were recorded during or after the procedure. Six months following the procedure, the patient remained in sinus rhythm, without recurrent episodes of AF, as documented by the absence of symptoms and four 24-h Holter recordings.

## Discussion and conclusions

The present study describes the case of successful PVI in a patient with DSI utilization of RMN and 3D-EAM system in combination with ICE-guided TSP, achieving similar procedure duration and X-ray exposure compared with patients without this unique anatomical variant. RF energy utilizing RMN system for PVI in AF patient with this anatomical variant has not been reported.

Dextrocardia is a term applied to all varieties of developmental malformations resulting in abnormal positioning of the heart. There are three types of situs: situs solitus (normal visceroatrial arrangement), situs inversus (mirror image of the normal visceroatrial arrangement), and situs ambiguous (visceroatrial isomerism), and the incidence rates of the three types of dextrocardia are similar [1]. DSI is characterized by the disordered anatomy of the atrium and altered IAS orientation. Regarding the relative position of the atrium, the RA changes from right anterosuperior to left anterosuperior relative to the LA, with a mirror image of the IAS. Moreover, the angle between the IAS and sagittal plane is approximately 45° in a normal heart, while the IAS in DSI is rotated 90° counterclockwise on the sagittal plane. The presence of dextrocardia poses significant technical challenges since catheter placement, TSP and manipulation of the catheters during PVI must be performed contrary to normal maneuvers, which might increase procedure duration and the risk of complications.

There are few case reports demonstrated that patients with DSI accompanied with AF have undergone successful PVI using manual cryoballoon [4], hotballoon [5], and manual-guided RF ablation [6, 7]. However, catheter and long sheath manipulations in manual-guided ablation are complex in cases of heart inversion, making it more difficult to achieve good catheter-to-tissue contact during PVI, especially in the left atrial ridge area [8]. Though preoperative cardiac CT was obtained to define the anatomic details before the procedure, two-dimensional information provided by X-ray images is insufficient in this abnormal anatomy during TSP and catheter ablation. Along with the advancement of 3D-EAM system and ICE application, we decided to perform catheter ablation using RMN system in combination with ICE-guided TSP for this patient, considering real-time images acquired with ICE can provide more anatomic details for operators and thereby ensure safety. In this case of DSI, TSP guided by ICE was quickly and safely performed. Additionally, RMN-guided AF catheter ablation offers precise and omni-directional catheter navigation with superior reach, resulting in lower fluoroscopy times and non-inferiority regarding periprocedural safety compared to the conventional manual guided approach [9, 10]. This robotic approach to AF ablation is routinely utilized in our center. The 3D EAM system and RMN system were particularly useful in locating the mirror-image PVs with accuracy and easily achieving PVI with robotic catheter navigation. As a result, the procedure time, RF time and fluoroscopic time were similar to patients without this unique anatomical variant as we reported in a previous study [11].

This case demonstrates that AF catheter ablation under the guidance of the RMN system using ICE is feasible and safe in a patient with DSI. Moreover, the combination of these technologies broadly facilitates treatment of patients with complex anatomy, while reducing the risk of complications.

## Data Availability

All data generated or analysed during this study are included in this published article.

## Abbreviation

AF: Atrial fibrillation
CS: Coronary sinus
CT: Computed tomography
DSI: Dextrocardia situs inversus
EAM: Electroanatomical mapping
FV: Femoral vein
IAS: Interatrial septum
ICE: Intracardiac echocardiograhy
LA: Left atrium
PV: Pulmonary vein
PVI: Pulmonary vein isolation
RA: Right atrium
RAO: Right anterior oblique
RF: Radiofrequency
RMN: Robotic magnetic navigation
TSP: Transseptal puncture
3D: Three-dimensional
